# Supplementary material for: Essential role of accessory subunit LYRM6 in the mechanism of mitochondrial complex I
Source: Nat Commun. 2020 Nov 26;11:6008. doi: 10.1038/s41467-020-19778-7 (PMC7693276; doi:10.1038/s41467-020-19778-7)
Supplement: Supplementary file 1 — Supplementary Information [file 41467_2020_19778_MOESM1_ESM.pdf]

Supplementary Information

Essential role of accessory subunit LYRM6 in the mechanism of mitochondrial complex I

Etienne Galemou Yoga, Kristian Parey, Amina Djurabekova, Outi Haapanen, Karin Siegmund, Klaus Zwicker, Vivek Sharma, Volker Zickermann and Heike Angerer

a

```
                                                                        α1
LYRM6_DICDI       --------------------------MS------SNMQAMKQMRPALVSLTQQQARRRCF   28
LYRM6_DROME       --------------------------MA----GREAVKRAVQQVRPILSVDREEARKRAL   30
LYRM6_DANRE       --------------------------MA-SSAATRTGSAAAKVVKPLFSRDLNEAKRRVR   33
LYRM6_XENLA       --------------------------MAA--SSAAATARAASAVKPILSRDLGEAKRRVR   32
LYRM6_CHICK       ---------------------------------MAVAGRGAAAVKPIFSRDLAEAKRRVR   27
LYRM6_HUMAN       MGKDIRPRSARAACKGVGLWSGCFGKMAGSGVRQAT-STASTFVKPIFSRDMNEAKRRVR   59
LYRM6_SHEEP       --------------NGVELGSDFLSKMAASGLRQA-AVAASTSVKPIFSRDMNEAKRRVR   45
LYRM6_MOUSE       ------------------------MAAAATGLRQAAAAAASTSVKPIFSRDLNEAKRRVR   36
LYRM6_PICPA       ---------------------------M--------TNPTAFAQTTRYVKDSTEMRTLVL   25
LYRM6_YARLI       --------------------------MA--------IIATAFAETVKFSGSKQELQKRTL   26
LYRM6_NEUSP       --------------------------MP--------ITPTKYAITTRQSANWSDAKRRVF   26
                                                                       : :

                      α1     α1´                α2                 α3
LYRM6_DICDI       KLYRNCIRSIPHLIQHYNLSYNMSEMRNRFRSNFVEFEEVTEKNQLDRLAFIGETELFDA   88
LYRM6_DROME       NLYKAWYRQIPYIVMDYDIPMTVEQCRDKLREEFVKHRNVTDIRVIDMLVIKGQMELKES   90
LYRM6_DANRE       ELYRAWYREAPNTVHAYQLDIGTQQCREKVREMFDRNRHVTDPRVIDMLVIKGKMELQET   93
LYRM6_XENLA       DLYRAWYREVPNSVHVFQLDITVKQGRD-VREMFQKNAHVTDLRVIDMLVIKGKMELEET   91
LYRM6_CHICK       ELYRAWYREVPNAVHLYQLDITAKQGRDKVREMFLKNAHVTDPRVIDMLVIKGKMDLQET   87
LYRM6_HUMAN       ELYRAWYREVPNTVHQFQLDITVKMGRDKVREMFMKNAHVTDPRVVDLLVIKGKIELEET   119
LYRM6_SHEEP       ELYRAWYREVPNTVHLFQLDISVKQGRDKVREMFKKNAHVTDPRVVDLLVIKGKMELEET   105
LYRM6_MOUSE       ELYRAWYREVPNTVHLMQLDITVKQGRDKVREMFMKNAHVTDPRVVDLLVIKGKMELQET   96
LYRM6_PICPA       NLYRRYLRHSRGFVNNYNLDIPASQVRTKIRQEFERQRFVKDLPLKNVLYMKAQMEFQEL   85
LYRM6_YARLI       ALYRQFLRGAPTFADLYEVQFSIPTIRTKIRQEFERHRFVDDLSIQNVLYAKGHMEYQEC   86
LYRM6_NEUSP       ALYRRWLRSTPEMQSMYSLPLPISVIRTRIRQEFERNRFVNKLPVVDVLLTKGHADYQET   86
                   **:   *         .:       *  .*. * .   * .    : *   .. :  :

                            α4                    α5
LYRM6_DICDI       MSLLKTRSHVVNYFDTQPVNAKTISESEKLLNNFFE---- 124
LYRM6_DROME       VEIWKQKGHIMRYWKESQD-----PKPTDFLSKFIQGVN- 124
LYRM6_DANRE       IQVWKQRTHVMRYFQETEA-----PRPADFLSKFYHGHNP 128
LYRM6_XENLA       IKVWKQRTHIMRYFHETET-----PRPTDFLSKFYAGQDP 126
LYRM6_CHICK       INVWKQRTHVMRYFHETET-----PRPKDFLSKFYEGHDP 122
LYRM6_HUMAN       IKVWKQRTHVMRFFHETEA-----PRPKDFLSKFYVGHDP 154
LYRM6_SHEEP       INVWKQRTHVMRFFHETEA-----PRPKDFLSKFYVGHDP 140
LYRM6_MOUSE       IKVWKQRTHVMRFFHETET-----PRPKDFLSKFYMGHDP 131
LYRM6_PICPA       VNFWKQQCHVMQYFESIDH-QNK-IKGDSFVQKFLKGAQ- 122
LYRM6_YARLI       INFWKQQAQFLKYFPEEDDIQGR-HQPSNFVDKFLKNRA- 124
LYRM6_NEUSP       MNFWRQTTHMMSYFNEESFRGAK-RLPSSFIDGFLQGRN- 124
                  :.. :   :.: ::              .::. *
```

b

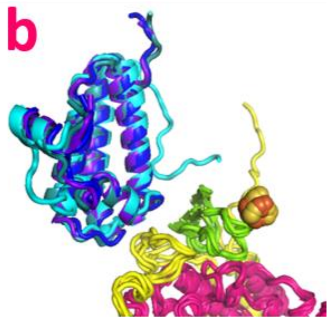

Supplementary Fig. 1: *Conservation of accessory subunit LYRM6.* (**a**) LYRM6 protein sequences from fungi and animals were aligned using CLUSTAL O multiple sequence alignment[1]. The LYR motif is highly conserved (bold). The sequences of the two loops studied in this work (highlighted in light grey) are moderately conserved. Selected residues of this study are shown in cyan and mutated residues $L42^{LYRM6}$, $Y43^{LYRM6}$ and $F89^{LYRM6}$ with DBQ activities <25% are highlighted in red, respectively (compare **Supplementary Table 1**). α-helices of *Y. lipolytica* LYRM6 are labelled (the segment labelled α1´ was modeled as loop in some complex I structures). (**b**) LYRM6 shows high structural conservation. Superimposed complex I structures (at position of NDUFS2) with LYRM6 from yeast (cyan, PDB 6RFR), mouse (blue, PDB 6G2J), sheep (dark teal, PDB 6QC5) and human (purple, PDB 5XTD). Selected substructures of central subunits ND1 (hot pink), ND3 (yellow) and NDUFS2 (green) interacting with LYRM6 are displayed.

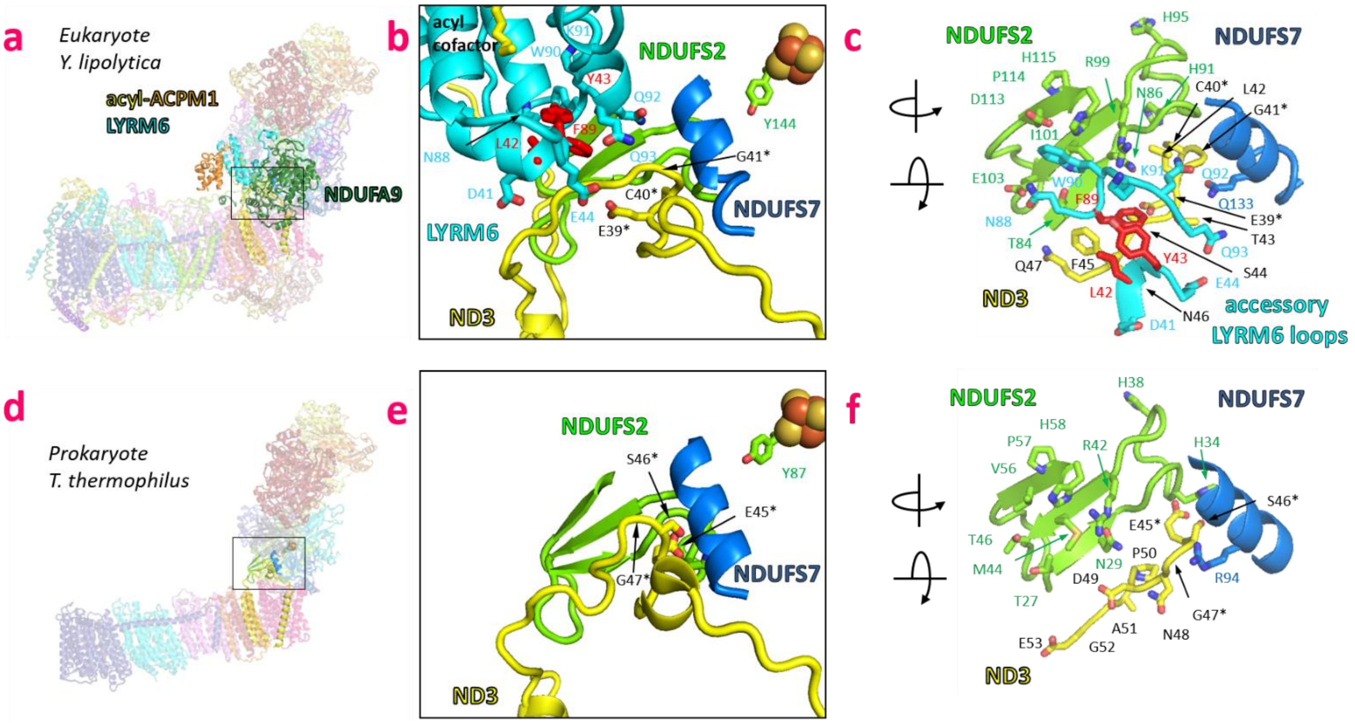

Supplementary Fig. 2: *Q/P module interface region in eukaryotic and prokaryotic complex I.* (**a**) Structure of *Y. lipolytica* WT complex I (PDB 6RFR) with accessory subunits LYRM6 (cyan), ACPM1 (orange) and NDUFA9 (forest). (**b,c**) close-up view of highlighted area in (**a**) without subunit NDUFA9, structural elements of central subunits NDUFS2 (green), NDUFS7 (marine) and ND3 (yellow) interact with LYRM6. Residues of LYRM6, NDUFS2, NDUFS7, ND3 and the acyl cofactor are shown in stick representation. Mutated LYRM6 residues with DBQ activities <25% are highlighted in red (see **Supplementary Table 1**). Residues $L42^{LYRM6}$, $Y43^{LYRM6}$ and $F89^{LYRM6}$ (red) of subunit LYRM6 form the central element of the LYRM6 accessory loop cluster. (**d**) Corresponding interface region in bacterial complex I (PDB 4HEA). (**e,f**) close-up view of highlighted area in (**d**), substructures of subunits ND3, NDUFS2 and NDUFS7. Residues $E45^{ND3}$ and $S46^{ND3}$ of *T. thermophilus* corresponding to $E39^{ND3}$ and $C40^{ND3}$ shown in (**b**) were suggested to contribute to an access pathway for substrate protons to the Q reduction site close to cluster N2[2]. *residues of highly conserved EXG^{ND3} motif (X = serine or cysteine).

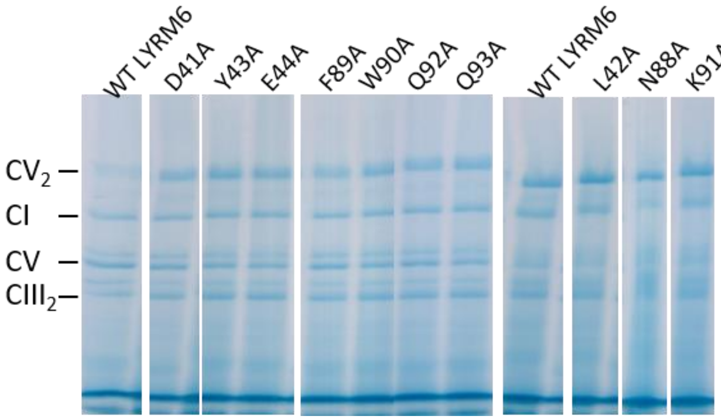

Supplementary Fig. 3: *Blue-Native (BN)-PAGE analysis of mitochondrial membranes from complex I LYRM6 mutants.* Coomassie-stained 4-16% BN-PAGE gel demonstrates that mitochondrial membranes of all LYRM6 mutants show assembled complex I and the results are in agreement with complex I contents determined by HAR activity (**Supplementary Table 1**). Image representative of n= 2 technical replicates. Uncropped gel in Source Data. CV_2, complex V dimer; CV, complex V monomer; CI, complex I; $CIII_2$, complex III dimer.

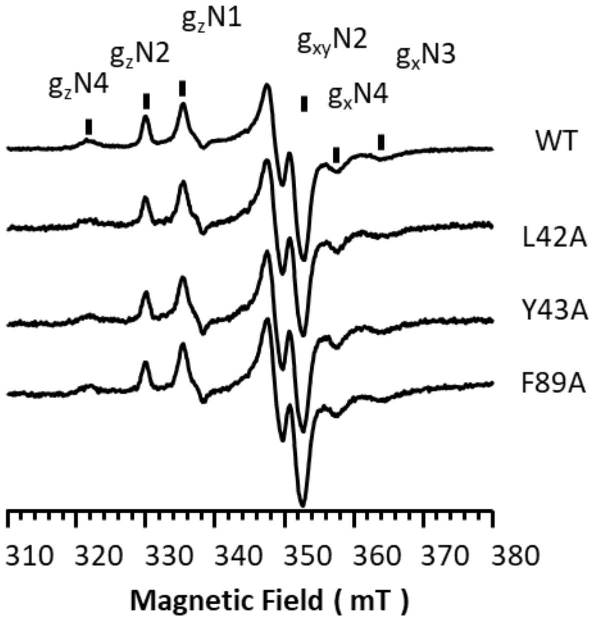

Supplementary Fig. 4: *Electron paramagnetic resonance analysis of purified complex I LYRM6 mutants.* EPR spectra of purified WT complex I and mutants L42A^{LYRM6}, Y43A^{LYRM6} and F89A^{LYRM6}. EPR spectra were recorded using the following parameters: microwave frequency, 9.47 GHz; microwave power, 1 mW; modulation amplitude, 0.64 mT; and modulation frequency, 100 kHz. Samples were reduced with NADH (2 mM), and the spectra were recorded at 12 K. Characteristic signal contributions of the four EPR visible Fe-S clusters of complex I (N1, N2, N3, N4) detectable under these conditions are indicated. For comparison, spectra were normalized on signal intensities of clusters N3 and N4. EPR spectra of mutants show same shape and intensity compared to WT and thus indicate intact Fe-S clusters and environment.

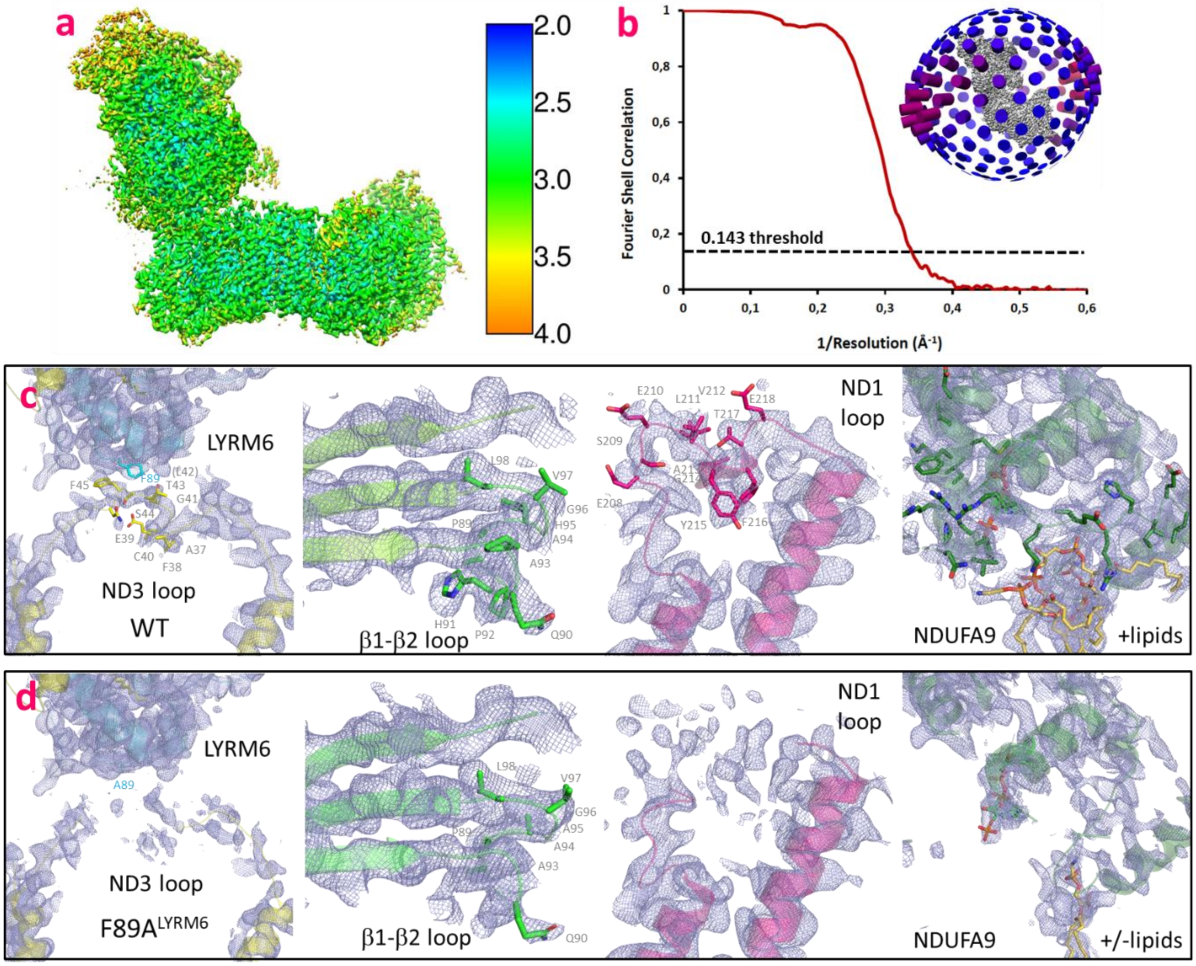

Supplementary Fig. 5: *Cryo-EM structure of inactive mutant F89A^{LYRM6}*. (**a**) Cryo-EM map of complex I analyzed with ResMap and colored by local resolution[3]. (**b**) FSC diagram for resolution estimation. Gold-standard FSC diagram between two separately refined half-maps shown in red. The map resolution is indicated by the point at which the curve falls below the threshold of 0.143[4]. Insert on the right: Angle distribution plot of particle images. The number of particles with their respective orientation is indicated by the length and color of the cylinders. (**c**) Density map of sections from subunits ND3/LYRM6, NDUFS2, ND1 and NDUFA9 of *Y. lipolytica* WT complex I at sigma level 1.5. (**d**) Corresponding density map of sections from subunits ND3/LYRM6, NDUFS2, ND1 and NDUFA9 of *Y. lipolytica* mutant F89A^{LYRM6} complex I at sigma level 1.5. Residues A37-Q47^{ND3} of THM1-2^{ND3} loop, H91^{S2} and P92^{S2} of subunit NDUFS2 (H95^{S2} was modelled as alanine), E208-E218^{ND1} of THM5-6^{ND1} loop, R219-K228^{A9}, R281-L289^{A9} and D342-N359^{A9} of NDUFA9 and lipids (stick representation) were not included into the model of F89A^{LYRM6} mutant.

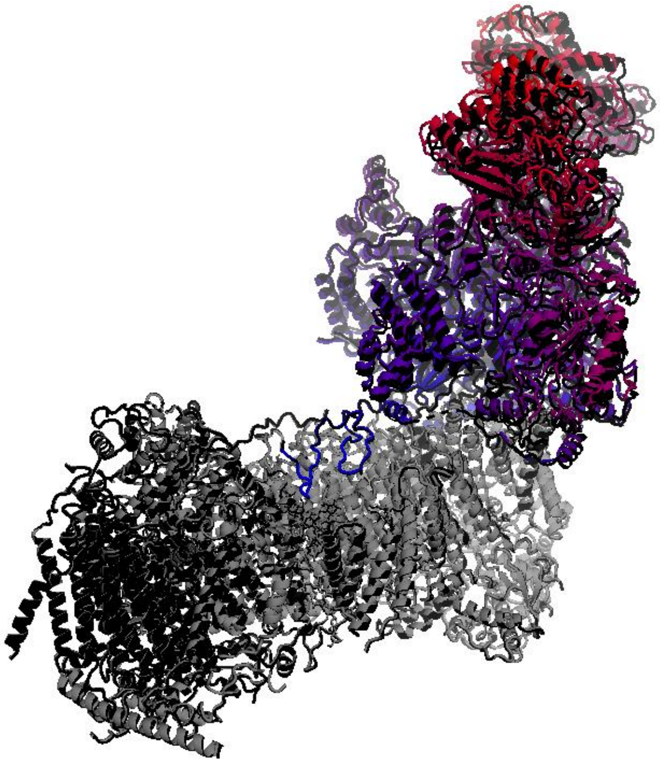

Supplementary Fig. 6: *Tilting of the matrix arm in F89A^{LYRM6} mutant.* Structures of mutant (grey) and WT (black) were superimposed at the position of membrane arm subunit ND4. Subunits of the matrix arm from F89A^{LYRM6} mutant are shown in color code (colored by root mean square deviation (RMSD), blue specifying the minimum pairwise RMSD and red indicating the maximum). Tilting of the matrix arm in the mutant was accompanied by disorder in C-terminal part of accessory subunit NDUFA9 and lipids at the membrane interface (**Supplementary Fig. 5d**).

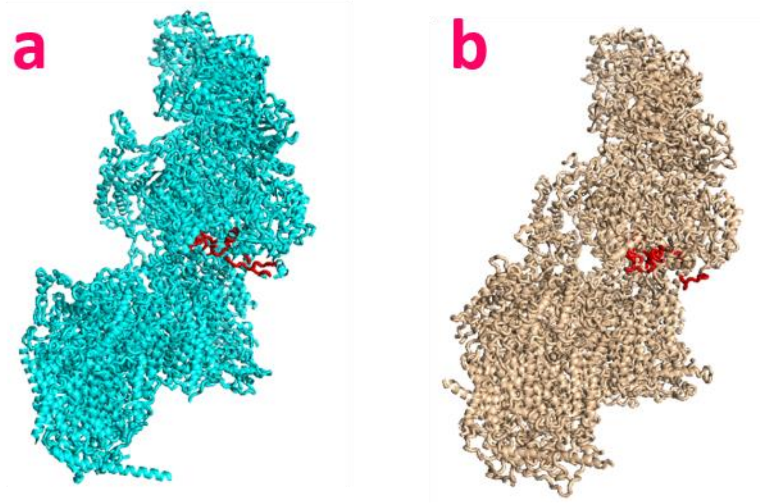

Supplementary Fig. 7: *Structural changes in F89A^{LYRM6} mutant compared to A/D transition of mouse complex I.* (**a**) F89A^{LYRM6} mutant (cyan) and (**b**) mouse complex I in D form (wheat, PDB 6G72). Red, densities observed in WT *Y. lipolytica* complex I (PDB 6RFR) and mouse A form (PDB 6G2J), however not resolved in F89A^{LYRM6} mutant and mouse D form, respectively.

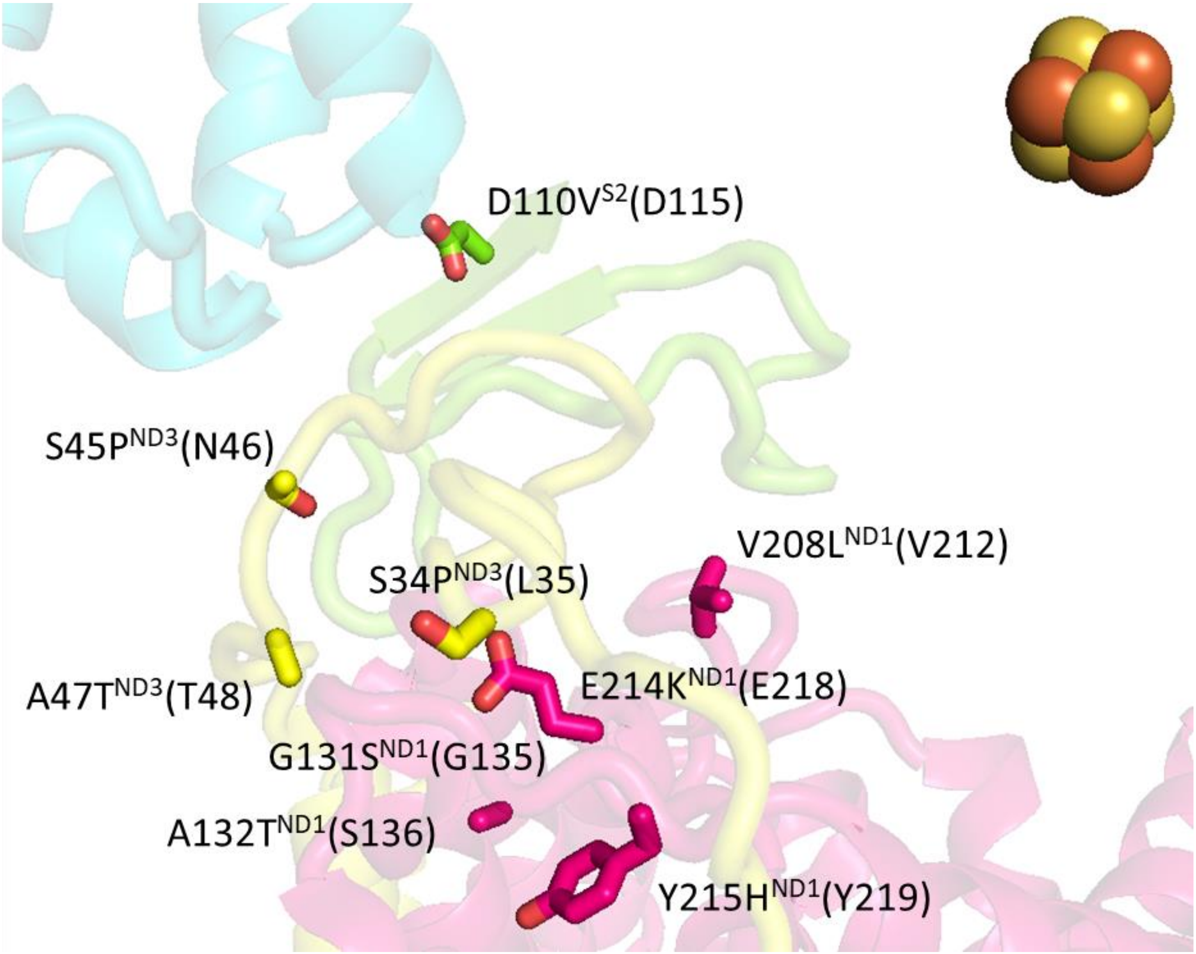

Supplementary Fig. 8. *Pathogenic mutations in human complex I.* Positions of selected residues with known pathogenic mutations (PDB 5XTD, human numbering) at the region of interest are highlighted in stick representation[5]. *Y. lipolytica* numbering is shown in parenthesis.

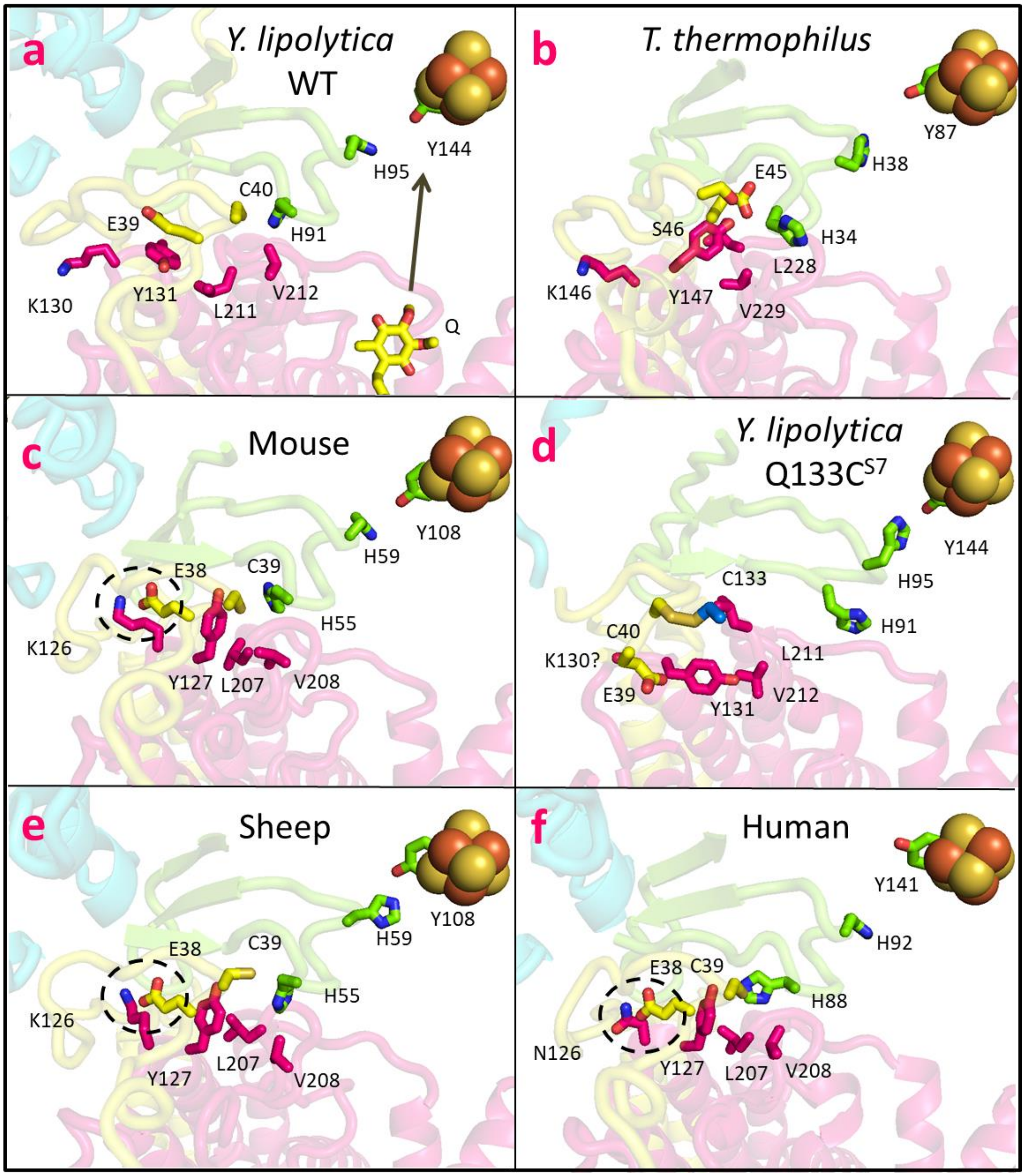

Supplementary Fig. 9: *Conformational variability of highly conserved E39^{ND3} of the TMH1-2^{ND3} loop.* Section of structures from (**a**) *Y. lipolytica* WT complex I (PDB 6RFR), (**b**) *T. thermophilus* complex I (PDB 4HEA), (**c**) mouse complex I in A form (PDB 6G2J), (**d**) *Y. lipolytica* mutant Q133C^{S7} with disulfide bond between C133^{S7} and C40^{ND3} (PDB 6H8K, K130^{ND1} modelled as alanine), (**e**) sheep complex I in closed state (PDB 6QC5) and (**f**) human complex I (PDB 5XTD); all structures superimposed at the position of subunit NDUFS2. The Q reduction site is indicated by an arrow in (**a**). The orientation of highly conserved mitochondrial ECG^{ND3} segment of TMH1-2^{ND3} loop is very similar in *Y. lipolytica* WT (D form), mouse complex I in A form and sheep in the closed state and human complex I. Structures demonstrate that the TMH1-2^{ND3} loop is flexible and occupies at least two different orientations. Also, noteworthy is the potential ion-pair formed between E45^{ND3}-H34^{S2} in *T. thermophilus* complex I (panel **b**) as part of the putative proton transfer pathway (see main text). Dashed circles highlight the salt bridge formed by residues K130^{ND1}-E39^{ND3} (in human complex I the lysine is replaced by an asparagine).

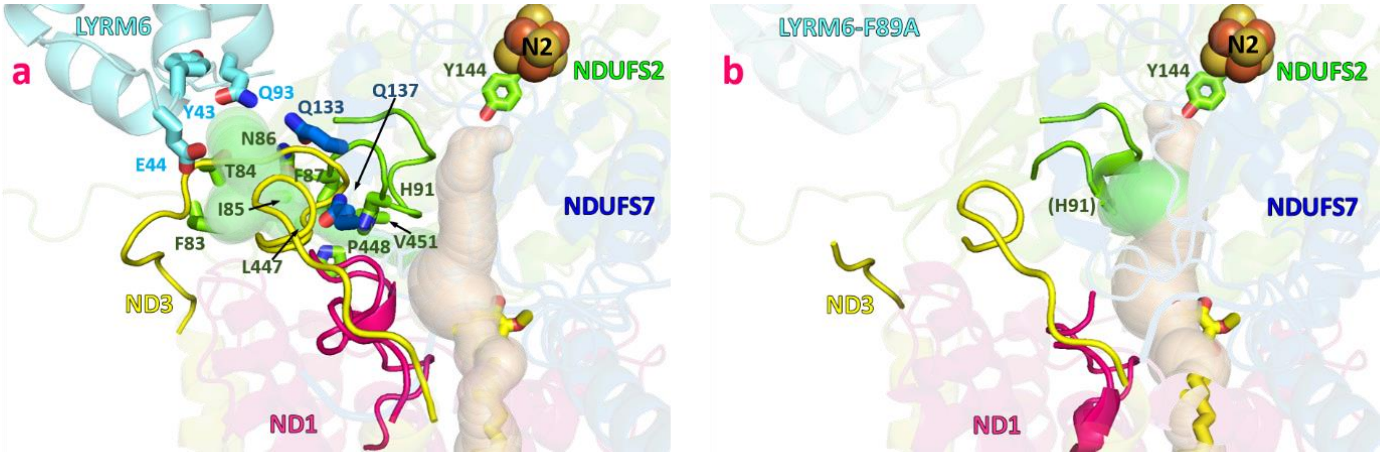

Supplementary Fig. 10: *CAVER analysis of WT Y. lipolytica complex I and F89A^{LYRM6} mutant.* (**a**) Residues of WT structure (PDB 6RFR) forming the CAVER tunnel (green) connecting the bulk N phase with the Q channel (beige) that were mutated to alanine by site-directed mutagenesis in this work are shown in stick presentation. Invariance of residues H91^{S2} and Y144^{S2} for complex I activity was reported previously[6,7] (**Supplementary Table 3**). Three residues of LYRM6 partially form the entrance of the tunnel (**Supplementary Table 3**). (**b**) CAVER analysis of the F89A^{LYRM6} mutant indicates disruption of the tunnel (green).

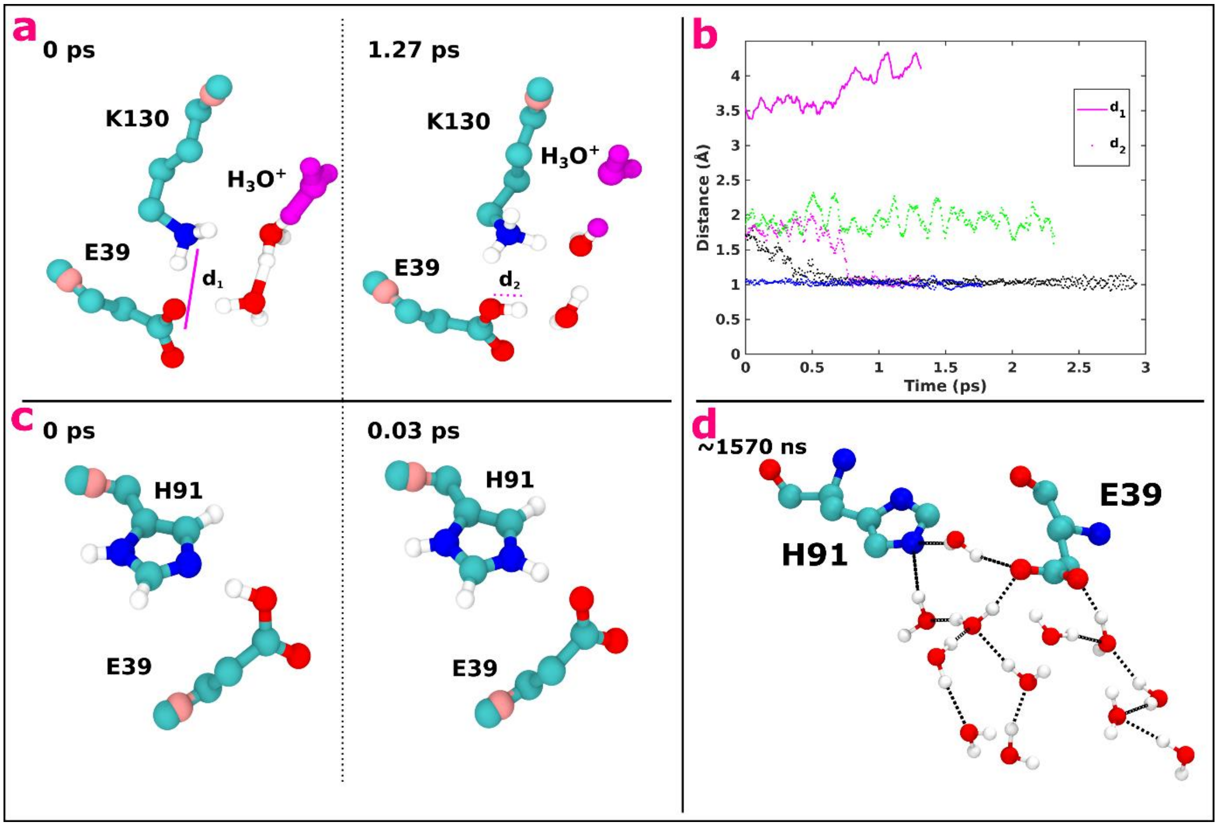

Supplementary Fig. 11: *Protonation dynamics in the channel.* (**a**) Proton transfer occurs from a modeled hydronium H_3O^+ (magenta) to anionic E39^{ND3} via a Grotthuss-like relay mechanism on water wires formed in classical MD simulations, shown as a simulation snapshot with selected water molecules (**d**). (**b**) Protonation of E39^{ND3} occurs together with the dissociation of ion-pair with K130^{ND1}. Distance d_1 is between the CD atom of E39^{ND3} and NZ atom of K130^{ND1}. Distance d_2 is between the OE1 atom of E39^{ND3} to its closest proton. In the plot, differently colored dotted lines (d_2) are from different QM/MM setups (see **Supplementary Table 6**). Magenta, Q1; black, Q2; blue, Q3 and green, Q4. The proton transfer does not occur in F89A^{LYRM6} mutant (green), whereas when E39^{ND3} is modeled neutral (blue), proton remains stable on it, suggesting it has higher proton affinity. (**c**) Rapid proton transfer occurs from E39^{ND3} to H91^{S2}, when the two are hydrogen bonding (setup Q5 in **Supplementary Table 6**, see also **Supplementary Fig. 9**). H-bonds are shown with black dotted lines in panel (**d**), and QM/MM link atoms are shown in pink in panels (**a**) and (**c**). Atom coloring; carbon – cyan, oxygen – red, nitrogen – blue and hydrogen – white.

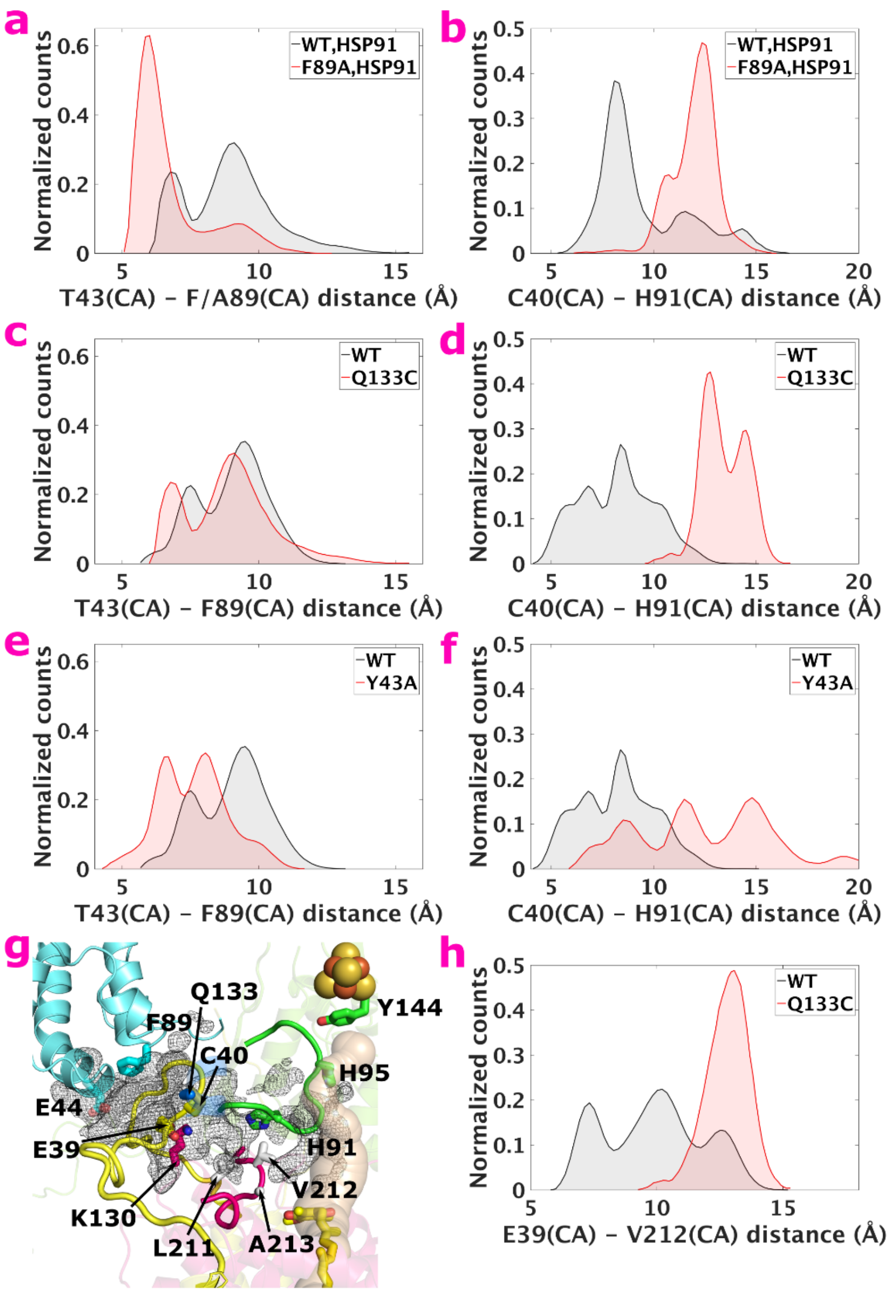

Supplementary Fig. 12: *Structural dynamics and hydration in WT and mutant MD simulation.* Displacement of ND3 loop segment (L42-S44^{ND3}) in F89A^{LYRM6} mutant simulations towards LYRM6 (**a**) and away from β1-β2^{S2} loop of NDUFS2 (**b**) with H91^{S2} modeled protonated (HSP91). Same distance data is plotted for Q133C^{S7} mutant MD simulations (**c** and **d**) and Y43A^{LYRM6} mutant (**e** and **f**). As expected, there is no major difference in T43^{ND3}-F89^{LYRM6} distance in WT and Q133C^{S7} MD simulations. Panel (**g**) shows continuous water occupancy in Q133C^{S7} simulations suggesting an "open channel". The isovalue of water occupancy map is 0.2, and entire data from all the simulation replicas was used. Panel (**h**) shows that the V212^{ND1}-E39^{ND3} "gate" is predominantly open in Q133C^{S7} simulations. The histograms shown are normalized so that the area under curve equals 1.

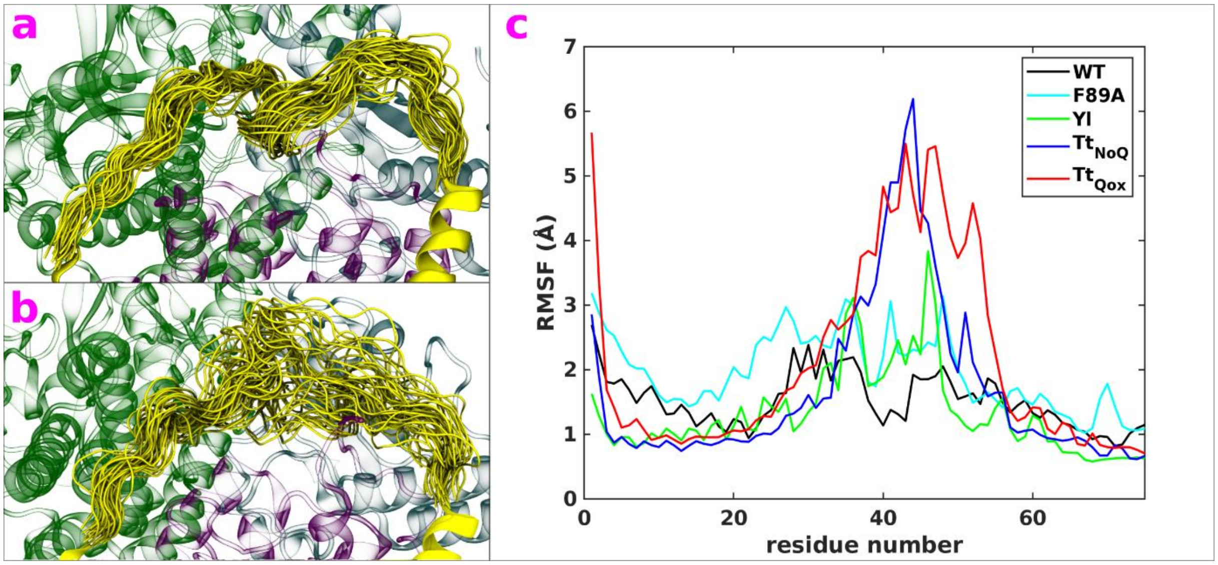

Supplementary Fig. 13: *Mobility of TMH1-2^{ND3} loop in bacterial and mitochondrial enzymes.* TMH1-2^{ND3} loop dynamics from *Y. lipolytica* complex I simulation setup 1 (**a**) and from *T. thermophilus* complex I setup 9 (**b**). The coordinates of ND3 loops are shown every 50 ns over entire simulation trajectories. (**c**) RMSF (root mean square fluctuation) of ND3 subunit for residues 1 to 75 from WT (setup 1), F89A (setup 2), large *Y. lipolytica* model system (setup 7), and the two *T. thermophilus* complex I simulations (setups 8 and 9). See **Supplementary Table 4** for simulation setups.

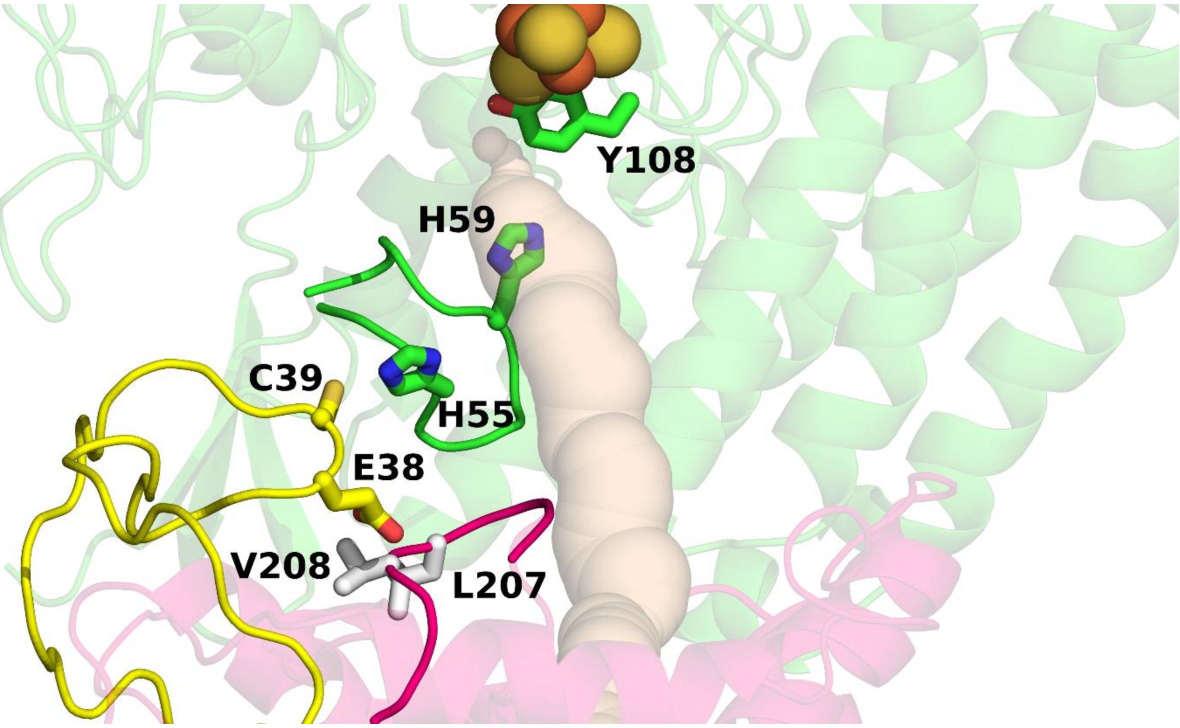

Supplementary Fig. 14: *Conserved segment from TMH5-6^{ND1} loop forms a bottleneck in the putative proton channel.* The conserved hydrophobic L207-V208^{ND1} residues block the channel in bovine complex I structure (PDB 5LC5) in agreement with our mutant MD simulation data (**Fig. 3c** and **i,** see also **Supplementary Table 5**). L207^{ND1} and V208^{ND1} (bovine complex I numbering) are homologous to L211^{ND1} and V212^{ND1} of *Y. lipolytica* complex I. Human V208L^{ND1} is a pathogenic mutation causing Leigh syndrome[8]. The figure was prepared after modeling and refining the missing sidechain positions in complex I structure (see also[9]).

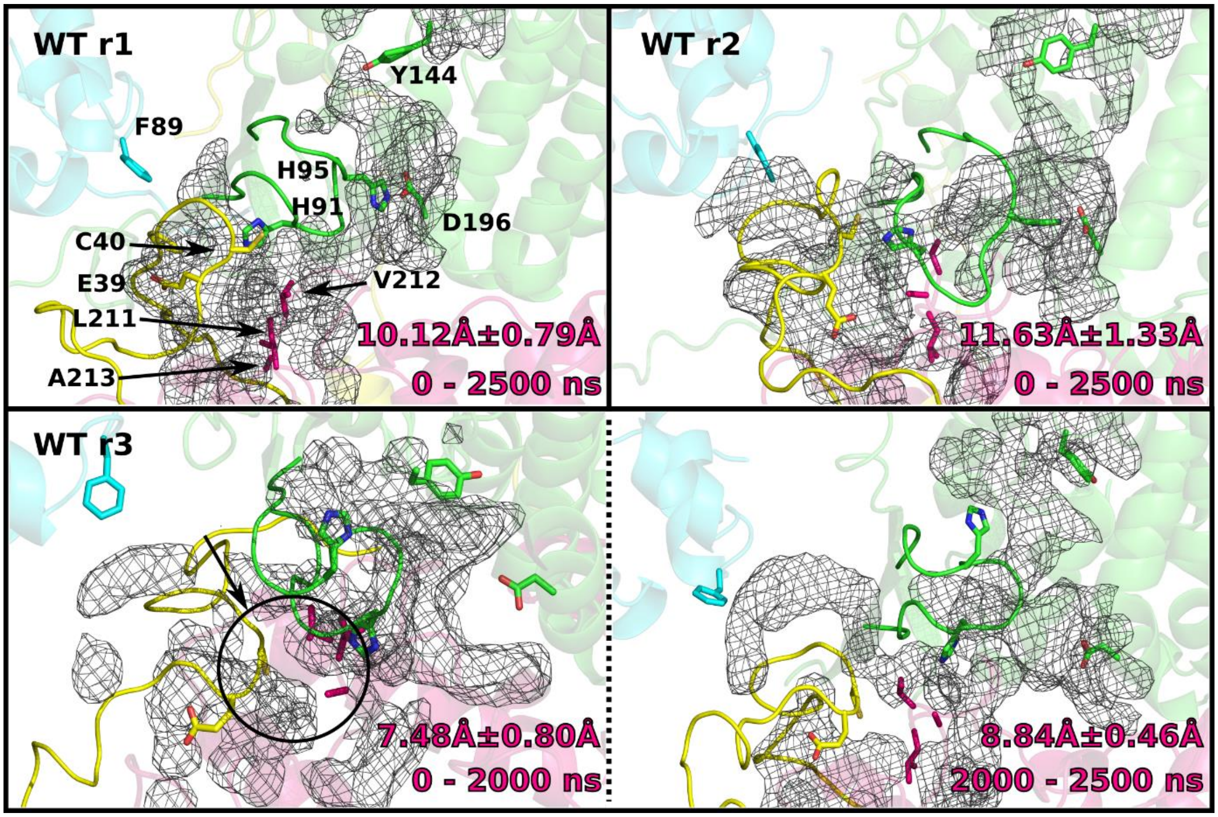

Supplementary Fig. 15: *Time dependent water occupancy (isovalue 0.2) analysis from WT simulations.* Water occupancy from WT runs (three replicas, r1-r3) are calculated based on the $E39^{ND3}$-$V212^{ND1}$ distance. The average distance between the Cα of $E39^{ND3}$ and $V212^{ND1}$ for the selected time frame is shown in each panel for each replica (lower right corner). The data show that in two of the three WT runs (r1 and r2), a continuous pathway is formed with larger $E39^{ND3}$-$V212^{ND1}$ distance. In simulation replica r3, there is a bottleneck in first 2 µs (black circle), but distance starts to increase and pathway starts to emerge in last 500 ns of simulation. See also **Fig. 3**.

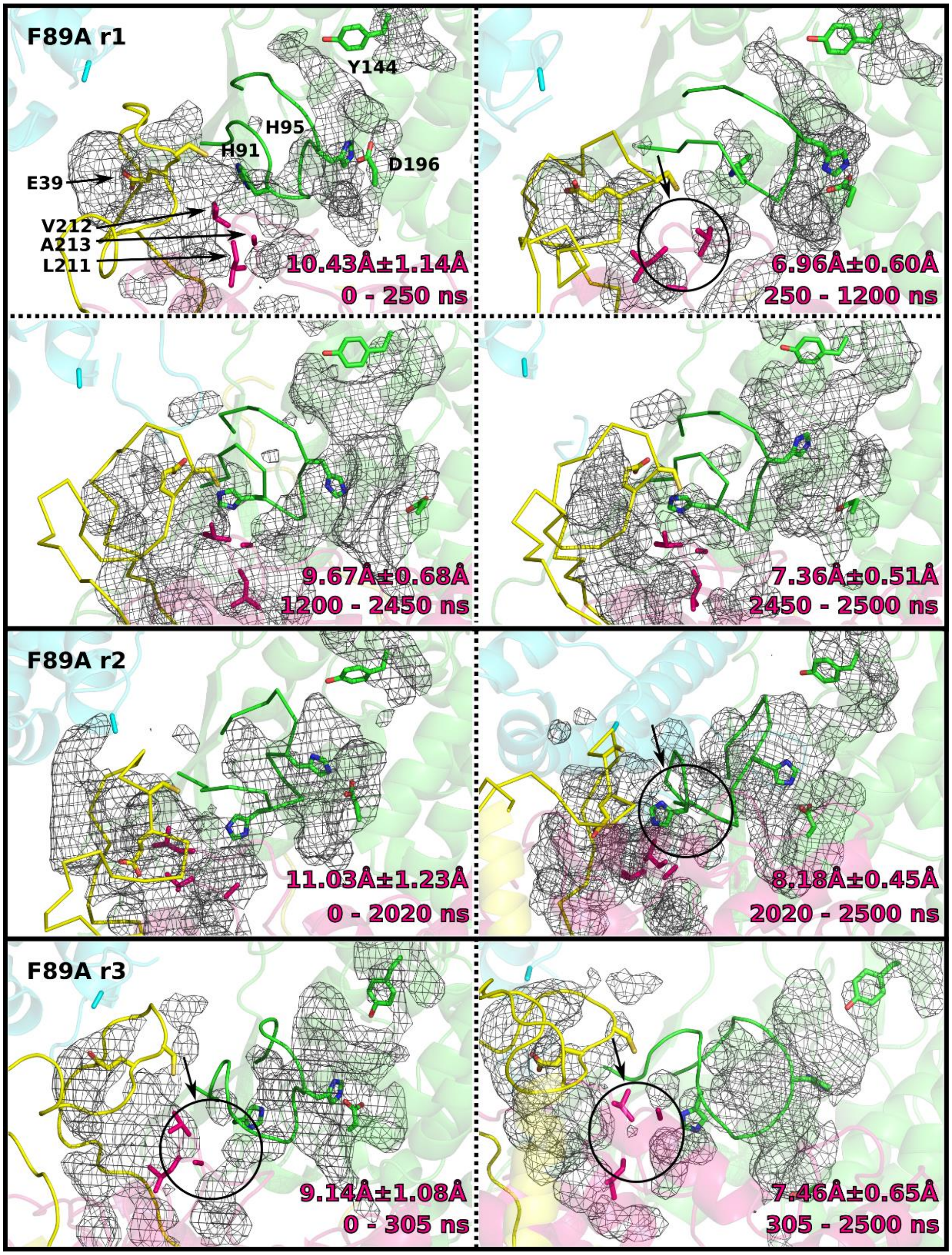

Supplementary Fig. 16: *Time dependent water occupancy (isovalue 0.2) analysis from F89A^{LYRM6} mutant simulations.* Water occupancy from F89A^{LYRM6} runs (three replicas, r1-r3) are calculated based on the E39^{ND3}-V212^{ND1} distance. The average distance between the Cα of E39^{ND3} and V212^{ND1} for the selected time frame is shown in each panel for each replica (lower right corner). The data shown for replica r3 reveals that the blockage is persistent for the entire simulation time, and in replica r1 for ca. 1.2 µs. These are marked by black circles, and the average V212^{ND1}-E39^{ND3} distance is lower in these cases (see also **Fig. 3**). In simulation replica r2, the path is hydrated initially, but at around 2 µs, the V212^{ND1}-E39^{ND3} distance started to decrease together with the reduction in hydration in the region. Also noteworthy is the flip of the sidechain of H91^{S2}, which is proximal to E39^{ND3}/C40^{ND3} when the pathway is more "open" (see main text).

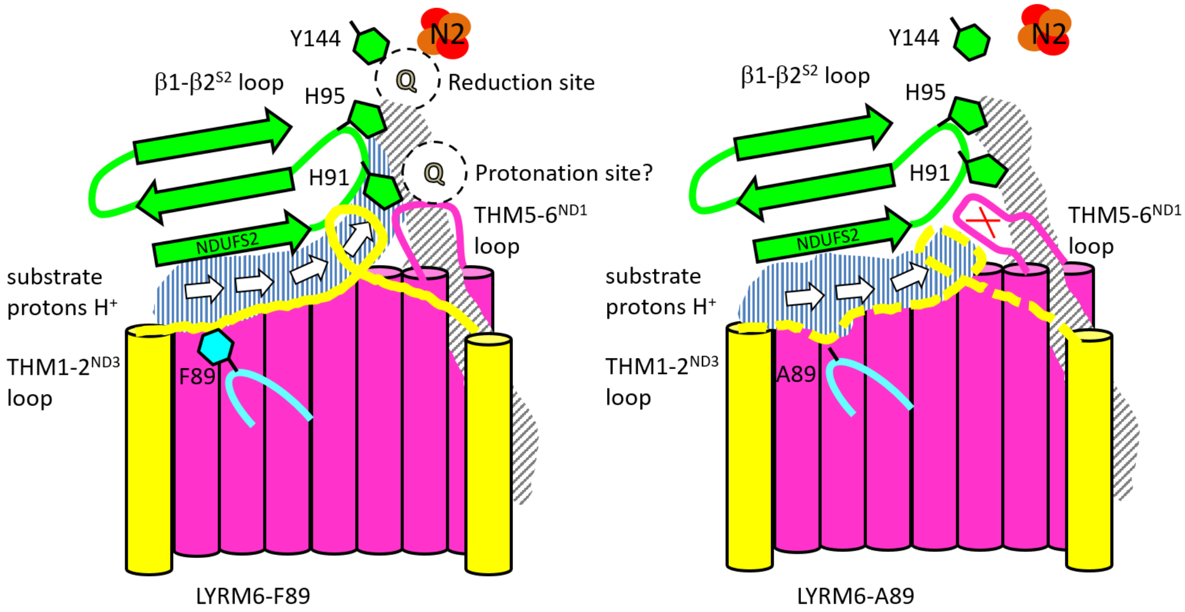

Supplementary Fig. 17: *Putative substrate proton pathway in mitochondrial complex I and its proposed functional mechanism.* Subunits ND1, ND3 and NDUFS2 form a putative pathway for substrate protons to the active site (left) that is blocked in mutant F89A^{LYRM6} (right). Q redox chemistry consumes two protons per QH_2 formed. The timing of refilling proton vacancies in the Q module via the channel identified in this work might play a key role in the proton pumping mechanism of complex I, but the exact identity of proton holes created upon two-electron reduction of Q is not known. It could be the anionic Q itself or amino acid residues left deprotonated after proton-coupled electron transfer (PCET) reaction[10]. Protonation of anionic Q at a site different from the electron accepting site near N2 is in agreement with the two-state stabilization change mechanism[11,12]. In other scenario, protonation of proton holes can occur via water molecules that have diffused into the channel only after departure of QH_2[13,14]. We suggest re-protonation from the bulk N phase is prevented in mutant F89A^{LYRM6} due to the structural rearrangements in the conserved loop cluster (β1-β2^{S2}, TMH5-6^{ND1} and TMH1-2^{ND3}). Due to the loss of bulky sidechain of F89 upon mutation to alanine, the ND3 loop rearranges rapidly, distancing from the β1-β2^{S2} loop of NDUFS2 (**Figures 2 and 3** and **Supplementary Figures 5 and 12**). We note that the ND3 and NDUFS2 loops have different conformations in complex I from *Y. lipolytica* and *T. thermophilus*, highlighting the dynamic behaviour of these domains and their functional role. Strictly conserved residues E39^{ND3} and H91^{S2} from these two loops are known to be catalytically important[6,15]. Their relative arrangement in the two complex I structures suggests that they may play a direct role in substrate proton transfer from the outside to the active site. Earlier, pKa calculations[16] have also shown a role of H91^{S2} (H34^{S2} in *T. thermophilus* complex I) in proton transfer (see **Supplementary Table 4** for WT and mutant models simulated with H91^{S2} protonated). The larger separation of (H91^{S2}) β1-β2^{S2} loop from (E39^{ND3}) TMH1-2^{ND3} loop in the mutant, together with the barrier caused by (L211-V212^{ND1}) TMH5-6^{ND1} loop will render substrate proton transfer unlikely. In contrast to predominantly closed proton transfer path in F89A^{LYRM6} mutant, extended opening of the channel as observed in mutant Q133C^{S7} simulations (**Supplementary Fig. 12**) might cause uncoupling of electron transfer and proton pumping by decreasing the lifetime of negatively charged reaction intermediates. This suggests that a coordinated movement of loop cluster is essential for the optimal timing of protonation of proton holes. Based on combined biochemical, structural and modeling data on WT and mutant enzymes, a new role of the conserved loop cluster is proposed, which may be relevant for the redox coupled proton pumping mechanism of complex I.

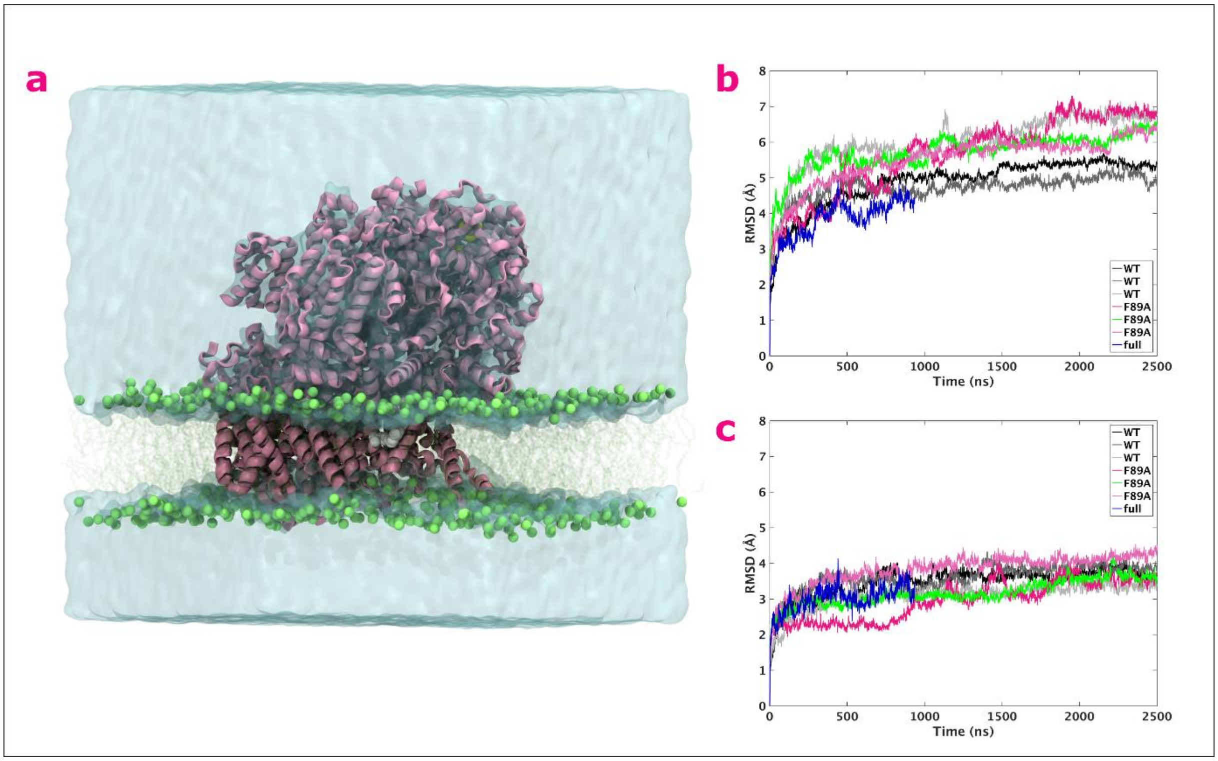

Supplementary Fig. 18: *The protein model system embedded in membrane-solvent environment and its dynamics.* (**a**) The protein is shown in pink ribbons, quinone in white spheres, lipid phosphorus atoms in light green spheres, lipid tails in transparent light green lines and water in turquoise surface. For clarity, ions are not shown. (**b**) RMSD (root-mean-square deviation) is plotted for the Cα atoms of the protein in WT and F89A^{LYRM6} simulations, and in large model system simulation (full - setup 7, see **Supplementary Table 4**). (**c**) Similarly, RMSD values for Cα atoms of Q module subunits NDUFS2, NDUFS7 and ND1. The data show that despite truncation, small model systems are stable. For panels (**b**) and (**c**), the terminus of NDUFS2 (residues 29 to 80) was omitted.

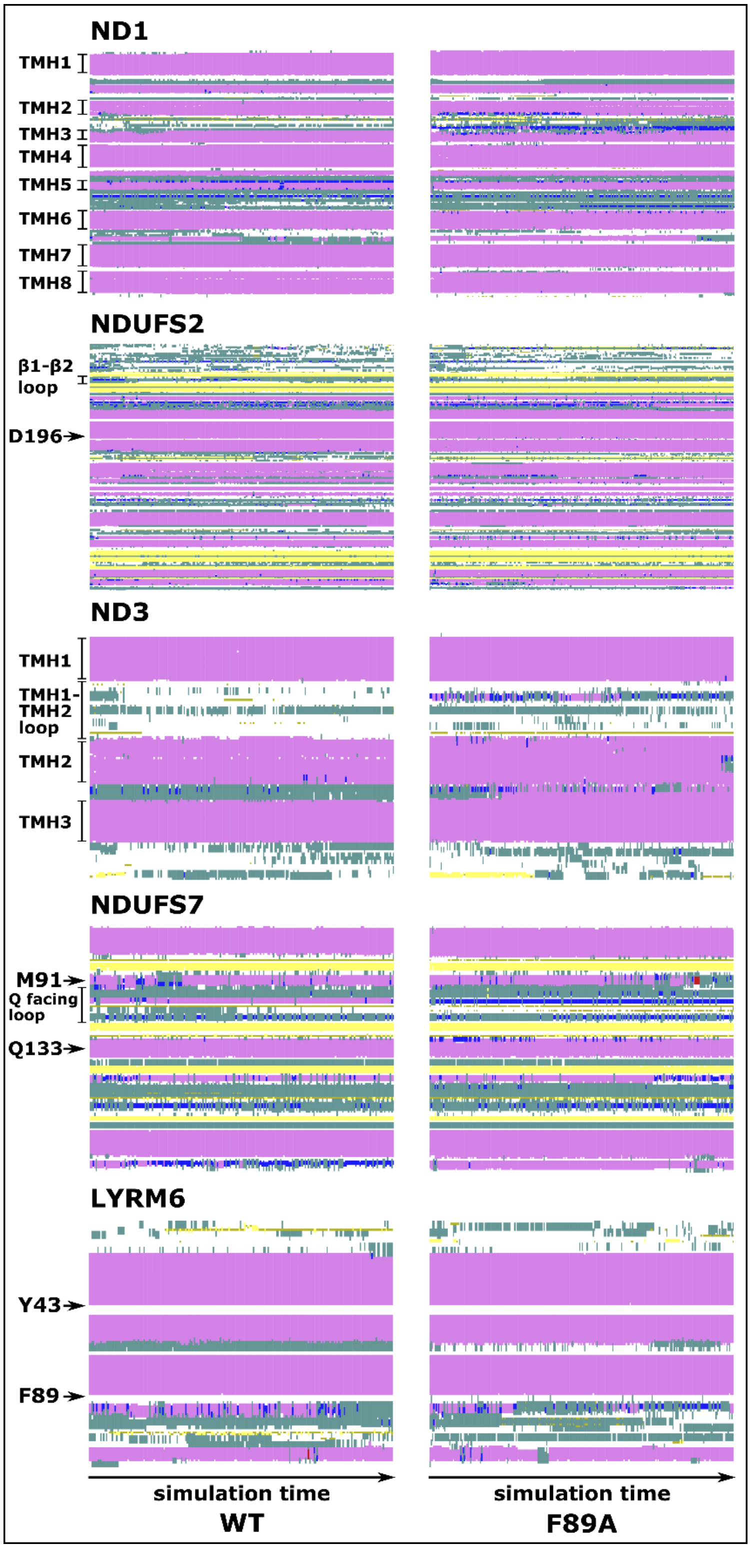

Supplementary Fig. 19: *Stability of secondary structure elements of key subunits.* Secondary structure profiles of WT and F89A^{LYRM6} mutant simulations (selected replicas) were calculated using VMD Timeline plugin for entire simulation length. Color coding; teal, turn; yellow, extended configuration (beta sheet); golden, isolated bridge; lilac, alpha helix; blue, 3-10-helix; red, Pi-helix and white, coil. Important structural features, such as transmembrane helices (TMH) and loops, and some key residues are indicated on the left.

Supplementary Table 1: *Complex I activities of accessory LYRM6 mutants.*

	Mitochondrial membranes	Mitochondrial membranes	Purified complex I
	Complex I content [%][a]	normalized DBQ activity [%][a]	normalized DBQ activity [%][b]
**WT-LYRM6**	100 ± 3	100 ± 8	100 ± 3
**D41A**	84 ± 11	26 ± 3	-
**L42A**	87 ± 13	24 ± 5	32 ± 4
**Y43A**	81 ± 11	14 ± 2	21 ± 1
**E44A**	98 ± 26	32 ± 4	-
**N88A**	83 ± 13	28 ± 2	-
**F89A**	82 ± 18	14 ± 1	12 ± 4
**W90A**	94 ± 1	32 ± 0	-
**K91A**	97 ± 5	78 ± 3	-
**Q92A**	95 ± 6	37 ± 5	-
**Q93A**	89 ± 7	83 ± 6	-

[a] Complex I content (WT^{LYRM6} HAR activity 100% = 1.4 U/mg) and normalized DBQ activity (WT^{LYRM6} DQA-sensitive DBQ activity 100% = 0.50 U/mg). Data are presented as mean values and errors represent standard deviations of at least three measurements of two biological replicas. Source data are provided as a Source Data file.

[b] WT complex I DBQ activity 100% = 21.5 ± 0.9 U/mg. Data are presented as mean values and errors represent standard deviations of at least three measurements of one sample. Source data are provided as a Source Data file.

Supplementary Table 2: *Cryo-EM data collection, refinement and validation statistics.*

	(EMDB-10711) (PDB 6Y79)
**Data collection and processing**	
**Magnification**	105,000x
**Voltage (kV)**	300
**Electron exposure (e–/Å^2)**	51.8
**Defocus range (μm)**	1.5-2.5
**Pixel size (Å)**	0.837
**Symmetry imposed**	C1
**Initial particle images (no.)**	479372
**Final particle images (no.)**	143203
**Map resolution (Å)**	2.96
**FSC threshold**	
**Map resolution range (Å)**	334.8-2.96
**Refinement**	
**Initial model used (PDB code)**	6RFR
**Map sharpening B factor (Å^2)**	-65
**Model composition**	
**Non-hydrogen atoms**	65173
**Protein residues**	7987
**Ligands**	44
**B factors (Å^2)**	
**Protein**	39.86
**Ligand**	40.15
**R.m.s. deviations**	
**Bond lengths (Å)**	0.012
**Bond angles (°)**	1.59
**Validation**	
**MolProbity score**	1.83
**Clashscore**	8.42
**Poor rotamers (%)**	0.37
**Ramachandran plot**	
**Favored (%)**	94.42
**Allowed (%)**	5.48
**Disallowed (%)**	0.1

Supplementary Table 3: *Tunnel analysis of Y. lipolytica WT complex I (PDB 6RFR).*

**NDUFS2**		**NDUFS7**		**ND1**	**ND3**	**LYRM6**		**NDUFA9**
**Caver Residue**[a]	**Mutagenesis, norm. CI activity [%]**[c]	**Caver Residue**[a]	**Mutagenesis, norm. CI activity [%]**[c]	**Caver Residue**[a]	**Caver residue**[a]	**Caver Residue**[a]	**Mutagenesis, norm. CI activity [%]**[c]	**Caver Residue**[a]
F83	F83A, 10 ± 2	**T80**		**K66**	N32	Y43	Y43A[g]	R208
T84	T84A, 19 ± 5	**F81**		**E67**	E33	***E44***	E44A[g]	K346
**I85**	I85A, 9 ± 4	**G82**		**N128**	K34	Q93	Q93A[g]	R349
**N86**	N86A, 9 ± 2	L83		**S129**	L35			
F87[b]	F87A, 4 ± 3	**A84**		**K130**	**G36**			
G88		**C85**		**Y131**	**A37**			
H91[b]	H91A, <5[d]	C86		**E206**	***F38***			
P92		**A87**		***S207***[b]	***E39***			
A93		V88		**E208**[b]	***C40***			
A94		E89		**S209**[b]	G41			
**H95**		M90		**E210**[b]	***L42***			
**G96**		**M91**		***L211***[b]	***T43***			
**V97**		S94		***V212***	***S44***			
**Y144**		I106		R297	***F45***			
M188		F107		**R302**	**N46**			
**S192**		R108			**Q47**			
**M195**		***A109***						
**D196**		**R112**	R112A, <10[e]					
V197		Q133	Q133A, 7 ± 1					
G198		D136	D136N, 17[f]					
L200		Q137	Q137A, 13 ± 2					
F203								
L447[b]	L447A, 8 ± 3							
P448	P448A, 89 ± 6							
**V451**[b]	V451A, 25 ± 4							
**V460**								

[a] Residues listed within 3.0 Å distance from the selected CAVER tunnel (**Fig. 3a**) starting from $Y144^{S2}$ are shown. The residues in italicized font are the ones selected to perform the average water occupancy calculations (**Fig. 3b and c**) by selecting water molecules that are within 4 Å from these residues. A large subset of residues (bold font) are found to be located in and around the calculated water occupancy map (**Fig. 3b**). Q channel residues are underlined. Grey, residues showing disorder in mutant $F89A^{LYRM6}$.

[b] residues with the narrowest section of the tunnel calculated by CAVER.

[c] normalized DQA-sensitive DBQ activities of mitochondrial membranes (WT^{NDUFS2} 100% = 0.54 ± 0.07 U/mg and WT^{NDUFS7} 100% = 0.60 ± 0.09 U/mg). Data are presented as mean values and errors represent standard deviations of at least three measurements of two biological replicas. Source data are provided as a Source Data file.

[d] previously reported[6]

[e] previously reported[17]

[f] previously reported[18], determined as NADH:nonyl-ubiquinone activity

[g] data shown in **Supplementary Table 1**.

Supplementary Table 4: *Model systems for classical MD simulations and their simulation lengths.*

System	WT/mutation[a]	Time (~ns) x simulation replicas
**1**	WT	2500 x 3
**2**	F89A^{LYRM6}	2500 x 3
**3**	Y43A^{LYRM6}	500 x 3
**4**	WT (H91^{S2} protonated)	500 x 2
**5**	F89A^{LYRM6} (H91^{S2} protonated)	500 x 2
**6**	Q133C^{S7} (disulfide bridge between C133^{S7} and C40^{ND3})	500 x 2
**7**	WT *(Y. lipolytica)*	1000 x 1
**8**	WT *(T. thermophilus* with Q10)	2000 x 1
**9**	WT *(T. thermophilus* without Q, H91^{S2} protonated)	2000 x 1

[a] *Yarrowia lipolytica* numbering

Supplementary Table 5: *Selected distances from available structures of complex I (CI) and related enzymes.*

Structure (PDB)	Remarks[a]	$T43^{ND3}$-$F89^{LYRM6}$	$C40^{ND3}$-$H91^{S2}$	$V212^{ND1}$-$E39^{ND3}$
6H8K	MCI *yeast*, $Q133C^{S7}$	-[c]	16.6	17.08
6RFR	MCI *yeast*	9.51	5.71	8.14
6YJ4	MCI *yeast*	8.60	11.67	9.54
6G2J	MCI *murine*	7.61[d]	6.43	9.64
5LC5	MCI *bovine*	10.45[d]	5.72	6.29
5LNK	MCI *ovine*	9.03[d]	25.06	20.50
6QBX	MCI *ovine*	7.77[d]	6.8	9.53
6QC5	MCI *ovine*	8.19[d]	6.95	9.98
5XTD	MCI *human*	8.42[d]	7.21	10.11
5XTH	MCI *human*	8.63[d]	7.36	10.11
6ZKO	MCI *ovine*	7.79[d]	6.74	9.59
6ZKG	MCI *ovine*	7.63[d]	6.55	9.32
6ZKK	MCI *ovine*	7.97[d]	6.54	9.60
6ZKC	MCI *ovine*	7.42[d]	6.44	9.30
4HEA	Bacterial CI	-[c]	-[c]	12.93
6HUM	Photosynthetic CI	-[c]	-[c]	10.05
6CFW	MBH[b]	-[c]	-[c]	12.65

[a] MCI, mitochondrial complex I

[b] MBH, membrane bound hydrogenase

[c] “-“ means either the atom/residue/subunit is not resolved or does not exist.

[d] Thr^{ND3}-Phe^{LYRM6} is replaced by Asp^{ND3}-Val^{LYRM6} in murine, bovine, ovine and human complex I.

Supplementary Table 6: *Quantum Mechanical/Molecular Mechanical (QM/MM) model systems and their simulation lengths. Selected residues in QM region are mentioned.*

System	WT/mutation	Time (~ps)
**Q1**	WT ($K130^{ND1}$-$E39^{ND3}$ ion pair)<br>(QM - $H91^{S2}$, $N86^{S2}$, $K130^{ND1}$, $V212^{ND1}$, $E39^{ND3}$, 6 H_2O, 1 H_3O^+)	1.32
**Q2**	WT (without $K130^{ND1}$-$E39^{ND3}$ ion pair)<br>(QM - $H91^{S2}$, $E39^{ND3}$, 8 H_2O, 1 H_3O^+)	2.93
**Q3**	WT ($E39^{ND3}$ neutral)<br>(QM - $H91^{S2}$, neutral $E39^{ND3}$, 8 H_2O)	1.32
**Q4**	$F89A^{LYRM6}$ mutant<br>(QM - $N86^{S2}$, $E39^{ND3}$, $V212^{ND1}$, 10 H_2O, 1 H_3O^+)	2.52
**Q5**	WT (hydrogen bond between $E39^{ND3}$ and $H91^{S2}$)<br>(QM – neutral $E39^{ND3}$, $H91^{S2}$, 7 H_2O)	0.35

Supplementary References

1 Sievers, F. & Higgins, D. G. Clustal Omega for making accurate alignments of many protein sequences. *Protein Sci* **27**, 135-145, doi:10.1002/pro.3290 (2018).

2 Jussupow, A., Di Luca, A. & Kaila, V. R. I. How cardiolipin modulates the dynamics of respiratory complex I. *Sci Adv* **5**, eaav1850, doi:10.1126/sciadv.aav1850 (2019).

3 Kucukelbir, A., Sigworth, F. J. & Tagare, H. D. Quantifying the local resolution of cryo-EM density maps. *Nat Methods* **11**, 63-65, doi:10.1038/nmeth.2727 (2014).

4 Rosenthal, P. B. & Henderson, R. Optimal determination of particle orientation, absolute hand, and contrast loss in single-particle electron cryomicroscopy. *J Mol Biol* **333**, 721-745, doi:10.1016/j.jmb.2003.07.013 (2003).

5 Fiedorczuk, K. & Sazanov, L. A. Mammalian Mitochondrial Complex I Structure and Disease-Causing Mutations. *Trends Cell Biol* **28**, 835-867, doi:10.1016/j.tcb.2018.06.006 (2018).

6 Grgic, L., Zwicker, K., Kashani-Poor, N., Kerscher, S. & Brandt, U. Functional significance of conserved histidines and arginines in the 49-kDa subunit of mitochondrial complex I. *J Biol Chem* **279**, 21193-21199, doi:10.1074/jbc.M313180200 (2004).

7 Tocilescu, M. A. *et al.* The role of a conserved tyrosine in the 49-kDa subunit of complex I for ubiquinone binding and reduction. *Biochimica et Biophysica Acta* **1797**, 625-632 (2010).

8 Wray, C. D. *et al.* A new mutation in MT-ND1 m.3928G>C p.V208L causes Leigh disease with infantile spasms. *Mitochondrion* **13**, 656-661, doi:10.1016/j.mito.2013.09.004 (2013).

9 Haapanen, O., Djurabekova, A. & Sharma, V. Role of Second Quinone Binding Site in Proton Pumping by Respiratory Complex I. *Front Chem* **7**, 221, doi:10.3389/fchem.2019.00221 (2019).

10 Sharma, V. *et al.* Redox-induced activation of the proton pump in the respiratory complex I. *Proc Natl Acad Sci U S A* **112**, 11571-11576, doi:10.1073/pnas.1503761112 (2015).

11 Brandt, U. A two-state stabilization-change mechanism for proton-pumping complex I. *Biochim Biophys Acta* **1807**, 1364-1369, doi:10.1016/j.bbabio.2011.04.006 (2011).

12 Parey, K. *et al.* Cryo-EM structure of respiratory complex I at work. *Elife* **7**, doi:10.7554/eLife.39213 [doi];39213 [pii] (2018).

13 Wikstrom, M., Sharma, V., Kaila, V. R., Hosler, J. P. & Hummer, G. New perspectives on proton pumping in cellular respiration. *Chem Rev* **115**, 2196-2221, doi:10.1021/cr500448t (2015).

14 Parey, K. *et al.* High-resolution cryo-EM structures of respiratory complex I: Mechanism, assembly, and disease. *Sci Adv* **5**, eaax9484, doi:10.1126/sciadv.aax9484 (2019).

15 Kao, M. C. *et al.* Functional roles of four conserved charged residues in the membrane domain subunit NuoA of the proton-translocating NADH-quinone oxidoreductase from Escherichia coli. *J Biol Chem* **279**, 32360-32366, doi:10.1074/jbc.M403885200 (2004).

16 Warnau, J. *et al.* Redox-coupled quinone dynamics in the respiratory complex I. *Proc Natl Acad Sci U S A* **115**, E8413-E8420, doi:10.1073/pnas.1805468115 (2018).

17 Galemou Yoga, E. *et al.* Mutations in a conserved loop in the PSST subunit of respiratory complex I affect ubiquinone binding and dynamics. *Biochim Biophys Acta Bioenerg* **1860**, 573-581, doi:10.1016/j.bbabio.2019.06.006 (2019).

18 Ahlers, P. M., Zwicker, K., Kerscher, S. & Brandt, U. Function of conserved acidic residues in the PSST homologue of complex I (NADH:ubiquinone oxidoreductase) from *Yarrowia lipolytica*. *J Biol Chem* **275**, 23577-23582, doi:10.1074/jbc.M002074200 (2000).
