# Supplementary material for: Essential role of accessory subunit LYRM6 in the mechanism of mitochondrial complex I
Source: Nat Commun. 2020 Nov 26;11:6008. doi: 10.1038/s41467-020-19778-7 (PMC7693276; doi:10.1038/s41467-020-19778-7)
Supplement: Supplementary file 3 — Reporting Summary [file 41467_2020_19778_MOESM3_ESM.pdf]

nature research

Corresponding author(s): Heike Angerer, Vivek Sharma

Last updated by author(s): Oct 26, 2020

Reporting Summary

Nature Research wishes to improve the reproducibility of the work that we publish. This form provides structure for consistency and transparency in reporting. For further information on Nature Research policies, see Authors & Referees and the Editorial Policy Checklist.

Statistics

For all statistical analyses, confirm that the following items are present in the figure legend, table legend, main text, or Methods section.

n/a	Confirmed	
☐	☒	The exact sample size (n) for each experimental group/condition, given as a discrete number and unit of measurement
☐	☒	A statement on whether measurements were taken from distinct samples or whether the same sample was measured repeatedly
☒	☐	The statistical test(s) used AND whether they are one- or two-sided<br>*Only common tests should be described solely by name; describe more complex techniques in the Methods section.*
☒	☐	A description of all covariates tested
☒	☐	A description of any assumptions or corrections, such as tests of normality and adjustment for multiple comparisons
☒	☐	A full description of the statistical parameters including central tendency (e.g. means) or other basic estimates (e.g. regression coefficient) AND variation (e.g. standard deviation) or associated estimates of uncertainty (e.g. confidence intervals)
☒	☐	For null hypothesis testing, the test statistic (e.g. F, t, r) with confidence intervals, effect sizes, degrees of freedom and P value noted<br>*Give P values as exact values whenever suitable.*
☒	☐	For Bayesian analysis, information on the choice of priors and Markov chain Monte Carlo settings
☒	☐	For hierarchical and complex designs, identification of the appropriate level for tests and full reporting of outcomes
☒	☐	Estimates of effect sizes (e.g. Cohen's d, Pearson's r), indicating how they were calculated

Our web collection on statistics for biologists contains articles on many of the points above.

Software and code

Policy information about availability of computer code

Data collection	Cryo-EM data were collected using EPU software (Thermo Fisher Scientific Inc., USA) on FEI Titan Krios microscope (Thermo Fisher Scientific Inc., USA) equipped with K3 Summit detector (Gatan, USA). To collect molecular dynamics simulation data, we used GROMACS software (2018.6 and 2020.2), which is open source. EPR data collection was performed using Bruker ESP 300e EPR software. Other software; QCHEM (5.0), CHARMM (41b1).
Data analysis	Dose-fractionated micrographs of Cryo-EM were subjected to motion correction and dose-weighting of frames by MotionCor2 (Zheng et al, 2017). Micrograph-based contrast transfer function (CTF) was determined by Gctf and the resulting images were used for further analysis with the software package RELION3.0 (Zivanov et al, 2018). Local resolution was estimated with ResMap (http://resmap.sourceforge.net). Bioinformatic tools COOT (Emsley et al, 2010), PyMOL 2.3 (Schrödinger, LLC) and CAVER 3.0 (Chovancova et al, 2012) were used for structural analyses. Simulation data analysis is achieved with GROMACS tools and Visual Molecular Dynamics (VMD 1.9.3 - 1.9.5), which is also available freely to academics. EPR analysis was performed using Bruker ESP 300e EPR software.

For manuscripts utilizing custom algorithms or software that are central to the research but not yet described in published literature, software must be made available to editors/reviewers. We strongly encourage code deposition in a community repository (e.g. GitHub). See the Nature Research guidelines for submitting code & software for further information.

Data

Policy information about availability of data

All manuscripts must include a data availability statement. This statement should provide the following information, where applicable:

- Accession codes, unique identifiers, or web links for publicly available datasets
- A list of figures that have associated raw data
- A description of any restrictions on data availability

The cryo-EM structures of complex I mutant F89ALYRM6 has been deposited in the PDB with PDB ID 6Y79 [https://doi.org/10.2210/pdb6Y79/pdb] (Cryo-EM structure of a respiratory complex I F89A mutant) and the respective cryo-EM maps in the EMDB under accession numbers EMD-10711 [https://www.ebi.ac.uk/pdbe/entry/emdb/EMD-10711] (Cryo-EM structure of a respiratory complex I F89A mutant). All data needed to evaluate the conclusions in the paper are present in

the paper and/or the Supplementary Information. Additional data related to this paper may be requested from the authors. Source data are provided with this paper.

Field-specific reporting

Please select the one below that is the best fit for your research. If you are not sure, read the appropriate sections before making your selection.

☒ Life sciences ☐ Behavioural & social sciences ☐ Ecological, evolutionary & environmental sciences

For a reference copy of the document with all sections, see nature.com/documents/nr-reporting-summary-flat.pdf

Life sciences study design

All studies must disclose on these points even when the disclosure is negative.

Sample size	We designed and produced 20 different yeast complex I mutants. In MD simulations sample sizes were determined based on our earlier works and experience, sufficient to obtain reliable results (e.g. Haapanen et al., Front. Chem., 2019). In cyro-EM of F89ALYRM6 mutant 3D classification with a previous cryo-EM map of WT Y. lipolytica complex I (Parey et al., Sci. Adv., 2019) as an initial reference was applied and the best 3D class of 143,203 particles was used for refinement. Sample size in EPR spectroscopy was optimized according S/N.
Data exclusions	No data were excluded from biochemical and computational experiments.
Replication	We analyzed at least duplicates of two biological replicas of yeast mutant mitochondrial membranes. EPR and activity were measured on single purified complex I samples. In MD simulations multiple simulation replicas were performed for different model systems. Overall, consistent results were obtained from different biochemical and simulation replicas.
Randomization	Not relevant to the experimental design.
Blinding	Not relevant to the experimental design.

Reporting for specific materials, systems and methods

We require information from authors about some types of materials, experimental systems and methods used in many studies. Here, indicate whether each material, system or method listed is relevant to your study. If you are not sure if a list item applies to your research, read the appropriate section before selecting a response.

Materials & experimental systems

n/a	Involved in the study	
☒	☐	Antibodies
☒	☐	Eukaryotic cell lines
☒	☐	Palaeontology
☐	☒	Animals and other organisms
☒	☐	Human research participants
☒	☐	Clinical data

Methods

n/a	Involved in the study	
☒	☐	ChIP-seq
☒	☐	Flow cytometry
☒	☐	MRI-based neuroimaging

Animals and other organisms

Policy information about studies involving animals; ARRIVE guidelines recommended for reporting animal research

Laboratory animals	The study did not involve animals. We used yeast Yarrowia lipolytica strains GB30, Dnb4m, Dnucm and Dnukm
Wild animals	Not applicable
Field-collected samples	Not applicable
Ethics oversight	Not applicable

Note that full information on the approval of the study protocol must also be provided in the manuscript.
